# Interaction Between Traffic-Related Air Pollution and Parkinson Disease Polygenic Risk Score

**DOI:** 10.1001/jamanetworkopen.2025.0854

**Published:** 2025-03-17

**Authors:** Dayoon Kwon, Kimberly C. Paul, Cynthia Kusters, Jun Wu, Jeff M. Bronstein, Christina M. Lill, Matthias Ketzel, Ole Raachou-Nielsen, Johnni Hansen, Beate Ritz

**Affiliations:** 1 Department of Epidemiology, Fielding School of Public Health, UCLA (University of California, Los Angeles), Los Angeles; 2 Department of Neurology, David Geffen School of Medicine, UCLA, Los Angeles; 3 Cousins Center for Psychoneuroimmunology, Semel Institute for Neuroscience and Human Behavior, UCLA, Los Angeles; 4 Department of Psychiatry and Biobehavioral Sciences, David Geffen School of Medicine, UCLA, Los Angeles; 5 Department of Environmental and Occupational Health, School of Population and Public Health, University of California, Irvine; 6 Institute of Epidemiology and Social Medicine, University of Münster, Münster, Germany.; 7 Ageing Epidemiology Research Unit, School of Public Health, Imperial College, London, United Kingdom; 8 Department of Environmental Science, Aarhus University, Roskilde, Denmark; 9 Global Centre for Clean Air Research, Department of Civil and Environmental Engineering, University of Surrey, Guildford, United Kingdom; 10 Danish Cancer Institute, Danish Cancer Society, Copenhagen, Denmark

## Abstract

**Importance:**

Genetic and environmental factors are linked to Parkinson disease (PD), but the role of genetic susceptibility in the association between traffic-related air pollution (TRAP) and PD remains unclear.

**Objective:**

To assess the gene-environment interaction between the polygenic risk score (PRS) for PD and long-term TRAP exposure and to estimate the joint effect with PD risk.

**Design, Setting, and Participants:**

This population-based case-control study used a meta-analytical assessment of studies conducted in central California and Denmark. The Parkinson Environment and Genes (PEG) study in California (June 1, 2000, to July 31, 2017) included 634 patients with PD and 733 controls; the Parkinson Disease in Denmark (PASIDA) study (January 1, 2006, to December 31, 2017) included 966 patients with PD and 1045 controls. Data were analyzed from July 1 to October 31, 2024.

**Exposures:**

PRS was computed by summing the effect estimates of well-known risk alleles from an existing genome-wide association study’s summary statistics using participants’ genetic arrays. TRAP exposure was estimated using dispersion models to calculate long-term exposure (10- or 15-year means with a 5-year lag) to traffic-related pollutants (represented by carbon monoxide [CO] levels) at participants’ residences.

**Main Outcomes and Measures:**

The main outcome was diagnosis of PD. Using multivariable logistic regression, PD risk was estimated from interactions between PRS (per SD) and TRAP exposure (per IQR), with joint effects based on low (quartiles 1-3) and high (quartile 4) exposure levels.

**Results:**

A total of 1600 patients with PD (mean [SD] age, 65.1 [9.9] years; 990 [61.9%] male) and 1778 controls (mean [SD] age, 64.5 [10.3] years; 992 [55.8%] male) were included. Meta-analytical estimates suggest that both higher PRS and increased TRAP exposure increased PD risk, with an interaction effect estimate of 1.06 (95% CI, 1.00-1.12). Joint effect analysis indicated that individuals with both high PRS and high TRAP exposure were at greatest risk of PD (odds ratio, 3.05; 95% CI, 2.23-4.19) compared with the reference group with a low PRS and low TRAP exposure, suggesting a synergistic effect.

**Conclusions and Relevance:**

In this gene-environment interaction study, a combination of long-term air pollution exposure and genetic susceptibility strongly contributed to the risk of developing PD. Widespread exposure to air pollution makes TRAP an important modifiable risk factor affecting large populations globally, particularly individuals with genetic vulnerability.

## Key Points

**Question:**

How are gene-environment interactions between the polygenic risk score (PRS) for Parkinson disease (PD) and long-term exposure to traffic-related air pollution (TRAP) associated with PD risk?

**Findings:**

In this case-control study with meta-analysis of 1600 cases and 1778 controls, higher PD-PRS and increased TRAP exposure were associated with increased PD risk, with those at high PRS and high TRAP exposure facing the greatest risk.

**Meaning:**

These findings suggest that the combination of genetic susceptibility to PD and long-term exposure to TRAP contributes to the risk of developing PD, underscoring the need for further research to explore the underlying biological mechanisms.

## Teaser

This case-control study assesses the interactions between the polygenic risk score for Parkinson disease and long-term exposure to traffic-related air pollution and their joint association with Parkinson disease risk.

## Introduction

Parkinson disease (PD) is one of the fastest-growing neurodegenerative disorders worldwide,^1,2,3^ extending beyond population aging.^1^ While monogenic forms of PD, caused by mutations in a single gene, are rare (3%-5% of patients),^2^ genetic risk variants at multiple loci contribute to 16% to 36% of heritable risk.^4^ The increasing prevalence and incidence of PD underscores the importance of exploring environmental risk factors in genetically susceptible individuals, and gene-environment interactions may provide insights into PD mechanisms.

Long-term exposure to a mixture of traffic-related air pollution (TRAP), typically represented by carbon monoxide (CO), nitrogen dioxide (NO_2_), and nitrogen oxide (NO_x_) levels, has been associated with an increased PD risk.^5^ Proposed mechanisms include neuronal toxic effects, systemic and central nervous system inflammation, and alterations in gut physiology and the microbiome.^5^ However, most prior studies have been limited by short exposure or follow-up periods (≤5 years) and insufficient exposure contrasts, unlike recent work that incorporates longer exposure periods.^6^ Since PD prodromal symptoms develop decades before clinical diagnosis, long-term TRAP exposure may contribute to disease progression or accelerate an already activated disease process.

Studies exploring gene-environment interactions in PD are limited, mostly relying on a single candidate gene approach.^7,8,9,10^ Findings from these studies have often not been replicated,^11^ and single genetic variants may not sufficiently capture the full spectrum of genetic susceptibility. The polygenic risk score (PRS),^12^ which aggregates single-nucleotide variants (SNVs; formerly single-nucleotide polymorphisms) associated with PD in a genome-wide association study (GWAS),^4^ has been shown to be associated with age at onset^13^ and cognitive and/or motor progression in PD.^14,15^ By using the PRS, we can incorporate multilocus genotypic variability and investigate gene-environment interactions with TRAP with enhanced statistical power.

A positive association has previously been reported between long-term TRAP exposure and PD risk in central California^16^ and Denmark.^17^ In the present study, we assess the interactions and joint effects of long-term TRAP exposure and genetic predisposition with PD risk using meta-analytical approaches to enhance statistical power while accounting for differences in environmental and genetic context. We hypothesize that TRAP interacts with the PRS, potentially modifying PD risk.

## Methods

All participants in this case-control study provided written informed consent. Study protocols were approved by the UCLA (University of California, Los Angeles) institutional review board, Danish Data Protection Agency, and Copenhagen Region ethics committee. This study is reported following the Strengthening the Reporting of Observational Studies in Epidemiology (STROBE) reporting guideline.

### Study Populations

The Parkinson Environment and Genes (PEG) study is a population-based case-control study conducted in central California. Participants were enrolled in 2 phases: PEG1 (June 1, 2000, to December 31, 2007) recruited through local neurological offices, PD support groups, and newspaper and radio announcements, and PEG2 (March 1, 2010, to July 31, 2017) used a pilot PD registry in California. Eligibility criteria included early-stage PD (within 5 years of diagnosis), a minimum 5-year residency in California, and absence of other neurological conditions or terminal illness. PD diagnoses were confirmed as primary PD by movement disorder specialists with in-person exams. Population controls were randomly selected (restricted to the cases’ age distribution) and contacted through mail, telephone, or direct home visits using Medicare and property tax records. The study included 634 cases and 733 controls (eFigure 1 in Supplement 1) with demographic (self-reported gender, race/ethnicity) and pesticide exposure data (eMethods in Supplement 1).^18^ Race and ethnicity groups included African American or Black, American Indian or Alaska Native, Asian, Latino, and White, reflecting the varying racial and ethnic backgrounds in PEG.

The Parkinson Disease in Denmark (PASIDA) study is a population-based case-control study. Patients with PD (aged ≥35 years) were identified from the Danish National Hospital Register (January 1, 1996, to December 31, 2009). Medically trained staff, supervised by a movement disorder specialist, reviewed medical records to confirm a primary PD diagnosis according to established criteria.^19,20,21^ This review included all available data from extensive neurological clinic records. Controls were randomly selected from the Danish Central Population Register, were alive at the date of diagnosis for the index patients, and had not received a PD diagnosis prior to the index date of their matched case. Eligible patients with PD needed to have survived until the study interview (January 1, 2008, to December 31, 2010), which made for many prevalent cases. To minimize potential survival bias, we only included patients (and their respective controls) diagnosed between 1998 and 2009, for a total of 966 cases and 1045 controls (eFigure 2 in Supplement 1) with information on potential confounding variables.^8,17^

### TRAP Exposure

For PEG, we estimated annual TRAP concentrations (1981-2016) using the California Line Source Dispersion Model, version 4,^22,23^ based on local traffic flow, vehicle composition, and meteorological data within a 1.5-km buffer zone around each residential address (eMethods in Supplement 1). Validation studies showed strong agreement between modeled and measured TRAP, with correlation coefficients (*r* > 0.92) near freeways.^23,24^ CO level was used as a proxy for local TRAP.^25,26^ Historically, from the 1980s to the early 2000s, CO has been considered a reliable marker of TRAP, as it is directly emitted from vehicle engines and is less affected by atmospheric chemistry compared with NO_2_ and NO_x_. These estimated CO levels did not account for nontraffic sources, including industry, commercial activities, residences, and urban background pollution outside the 1.5-km buffer zone.

The long-term exposure period was selected based on the longest available TRAP data, ensuring a comprehensive analysis of long-term effects. For PEG, long-term TRAP exposure was calculated as the mean yearly concentrations during the decade before the PD diagnosis for cases and the interview year for controls. To account for PD diagnosis latency, a 5-year lag was incorporated (ie, the 10-year exposure period starts 15 years before the index date). Participants with at least 60% of their residential history known within this time frame were included. For approximately 9% of cases and controls, gaps in residential history were filled by calculating mean concentrations from adjacent years. No statistically significant differences were found in demographic or other characteristics between participants with complete and incomplete residential histories.

For PASIDA, residential histories were retrieved from the Central Population Registry dating back to 1971. Yearly TRAP (CO, NO_2_, and NO_x_) levels were estimated using the Danish DEHM/UBM/AirGIS (Danish Eulerian Hemispheric Model, Urban Background Model, and Geographical Information System–Based Air Pollution and Human Exposure Modeling System) dispersion model,^27^ which combines local traffic pollution with urban and regional background levels. Validation of this model against measured data in Copenhagen showed strong correlations (*r* = 0.90 for NO_2_ and *r* = 0.88 for NOx).^19,28,29^ Traffic-related pollutants (CO, NO_2_, and NO_x_) were all correlated (*r* = 0.82-0.93) (eTable 1 in Supplement 1). For consistency with PEG, we focused on CO as a proxy for TRAP. Given the longer available TRAP data for PASIDA, mean yearly concentrations were calculated for a 15-year period starting 20 years before the index year (the year of PD diagnosis for cases and matched controls), with a 5-year lag. Participants with at least 60% of their residential history within this time frame were included, with missing data for about 2% of cases and controls replaced by mean concentrations from adjacent years.

### Genotyping and PRS

Genotyping was performed using a commercially available kit (Global Screening Array, version 1; Illumina Inc) as previously described.^30,31^ The initial data processing, including clustering and genotype calling, have been previously described in detail^30,31^ and are provided in the eMethods in Supplement 1.

Of the 90 SNVs previously identified as PD risk loci in the GWAS by Nalls et al,^4^ 86 SNVs were available in both PEG and PASIDA and were used for the PD-PRS calculation. The PRS was generated by aggregating the cumulative estimated effects of these SNVs using a weighted-allele approach. An alternate PRS was calculated using a more stringent linkage disequilibrium clumping threshold (*r*^2^ = 0.001) (eMethods in Supplement 1), resulting in a PRS with 76 instead of 86 SNVs.

Because the PRS was derived from European-ancestry GWAS data,^4^ its applicability is most relevant to individuals of European ancestry due to differences in allele frequencies and linkage disequilibrium patterns across populations. Although PEG includes participants of various racial and ethnic backgrounds, the majority are of European ancestry, making this PRS appropriate for most of the cohort. However, its use in non-European participants may be limited, which we acknowledge as a study limitation.

### Statistical Analysis

We conducted separate analyses for the PD-PRS and TRAP exposure with PD in the PEG and PASIDA studies using unconditional logistic regression to calculate multivariable-adjusted odds ratios (ORs) and 95% CIs. The PRS (per SD) and TRAP exposure (per IQR) were treated as continuous variables or categorized into binary variables according to quartiles (low, quartiles 1-3; high, quartile 4). Statistical significance was assessed using 95% CIs for ORs. All statistical analyses were performed from July 1 to October 31, 2024, using R, version 3.5.1 (R Project for Statistical Computing).

We evaluated the independence of the PD-PRS and TRAP exposure using Spearman rank correlation in both studies. Crude unadjusted models can be compared with models adjusted for confounders, which were selected based on directed acyclic graphs (eFigure 3 in Supplement 1) that drew from prior research^16,17^ and reflect data availability. For PEG, models were adjusted for age, gender, years of education, study wave (PEG1 vs PEG2), pesticide exposure (total pesticide count within 500 m of each participant’s residence during the TRAP exposure period), smoking status (never vs ever), family history of PD, job history of air pollution–related exposures (eg, truck or bus drivers, traffic police, and asphalt workers), and the first 3 principal components of genetic ancestry. For PASIDA, adjustments included age, gender, educational level (basic, vocational, and higher), smoking status, family history of PD, job history, and the first 3 principal components of genetic ancestry, which capture relevant population-level genetic variation.

We assessed the marginal effects of the PD-PRS and long-term TRAP exposure on PD risk and included cross-product terms for PRS and TRAP to examine multiplicative interactions. We also estimated joint effects by creating mutually exclusive strata based on the cross-classification of PRS and TRAP categories. The reference group consisted of individuals with low PRS and low TRAP exposure. To estimate the expected joint effect with the multiplicative scale, we multiplied the individual effects of high PRS and high TRAP exposure generated in a model without an interaction term and compared the result with the observed joint effect. The PRS was further categorized into 3 levels (low: quartile 1; intermediate: quartiles 2-3; high: quartile 4) to examine a potential risk gradient for genetic susceptibility interactions with TRAP. Based on sample size considerations and to ensure interpretability of our results, we dichotomized TRAP exposure into low (quartiles 1-3) and high (quartile 4) categories.

Last, we conducted meta-analyses to combine results from both studies, calculating pooled estimates and 95% CIs. Heterogeneity across the studies was assessed using the Cochran *Q* test and the *I*^2^ statistics. Since no statistically significant heterogeneity was detected (interaction term: *Q* = 0.41; *P* = .52; *I*^2^ = 0) (eTable 2 in Supplement 1) and results from fixed- and random-effects models were basically identical, we reported only fixed-effect models.

In sensitivity analyses, we evaluated the more stringent alternate PD-PRS with 76 SNVs. We also conducted analyses restricted to participants with complete residential address histories. For PEG, we additionally adjusted for neighborhood socioeconomic status to account for potential confounding. For PASIDA, we conducted additional analyses using a 10-year exposure period with a 5-year lag to match the PEG exposure period length and restricted analyses to incident PD cases diagnosed between 2006 and 2009 to minimize potential survival bias or confounding from longer disease duration. Nonlinearity was tested using splines with 3 knots.

## Results

### Characteristics of Study Participants

We included a total of 1600 patients with PD (mean [SD] age, 65.1 [9.9] years; 610 [38.1%] female and 990 [61.9%] male) and 1778 controls (mean [SD] age, 64.5 [10.3] years; 786 [44.2%] female and 992 [55.8%] male). PEG included 1367 participants (634 PD cases and 733 controls) (Table 1) with a mean (SD) age of 66.7 (11.2) years (623 [45.6%] female and 744 [54.4%] male; 27 [2.0%] African American, 64 [4.7%] American Indian or Alaska Native, 37 [2.7%] Asian, 263 [19.2%] Latino, and 976 [71.4%] White). PEG participants were mostly nonsmokers (1255 [91.8%]) and had a mean (SD) of 13.7 (4.2) years of education. The study had a higher proportion of male patients with PD (398 [62.8%]) than male controls (346 [47.2%]), reflecting the overall gender distribution in the source population.

**Table 1.** Characteristics of the PEG and PASIDA Study Participants

Characteristic	No. (%) of participants			
	PEG		PASIDA	
	Cases (n = 634)	Controls (n = 733)	Cases (n = 966)	Controls (n = 1045)
Age, mean (SD), y	67.6 (10.7)	66.0 (11.5)	63.4 (8.9)	63.4 (9.2)
Gender				
Female	236 (37.2)	387 (52.8)	374 (38.7)	399 (38.2)
Male	398 (62.8)	346 (47.2)	592 (61.3)	646 (61.8)
Race and ethnicity				
African American	4 (0.6)	23 (3.1)	NA	NA
American Indian or Alaska Native	22 (3.5)	42 (5.7)	NA	NA
Asian	16 (2.5)	21 (2.9)	NA	NA
Latino	111 (17.5)	152 (20.7)	NA	NA
White	481 (75.9)	495 (67.5)	NA	NA
Educational attainment^a^				
Mean (SD), y	13.5 (4.4)	13.8 (4.0)	NA	NA
Basic	NA	NA	217 (22.5)	225 (21.5)
Vocational training	NA	NA	480 (49.7)	569 (54.4)
Higher	NA	NA	269 (27.8)	251 (24.0)
Smoking status				
Never	347 (54.7)	354 (48.3)	493 (51.0)	405 (38.8)
Former	262 (41.3)	292 (39.8)	398 (41.2)	447 (42.8)
Current	25 (3.9)	87 (11.9)	75 (7.8)	193 (18.5)
Study wave				
PEG1	292 (46.1)	308 (42.0)	NA	NA
PEG2	342 (53.9)	425 (58.0)	NA	NA
Place of residence				
Copenhagen and suburbs	NA	NA	201 (20.8)	312 (29.9)
Provincial cities	NA	NA	617 (63.9)	527 (50.4)
Rural	NA	NA	148 (15.3)	206 (19.7)
PD family history				
No	494 (77.9)	677 (92.4)	840 (87.0)	990 (94.7)
Yes	140 (22.1)	56 (7.6)	126 (13.0)	55 (5.3)
Air pollution–related job history^b^				
No	485 (76.5)	627 (85.5)	830 (85.9)	883 (84.5)
Yes	149 (23.5)	106 (14.5)	136 (14.1)	162 (15.5)

Abbreviations: NA, not applicable; PASIDA, Parkinson Disease in Denmark; PD, Parkinson disease; PEG, Parkinson Environment and Genes.

^a^ Educational levels for PASIDA participants are categorized as follows: basic education includes 7 to 12 years; vocational training, 10 to 12 years; and higher education, 13 or more years.

^b^ Includes any history of employment as a traffic police officer, gas station attendant, mechanic, asphalt worker, or a professional truck, tractor, or bus driver.

PASIDA included 2011 participants (966 cases and 1045 controls) with a mean (SD) age of 63.4 (9.0) years; 773 (38.4%) were female and 1238 (61.6%) were male. Most were nonsmokers or former smokers (1743 [86.7%]). Nearly three-quarters had completed basic and vocational education (1491 [74.1%]), and more than half resided in provincial cities within Denmark (1144 [56.9%]). There was no correlation between the PD-PRS and TRAP exposure in either PEG (ρ = 0.02; *P* = .38) or PASIDA (ρ = 0.03; *P* = .19), confirming the independence of genetic and environmental factors (eFigure 4 in Supplement 1).

### Results for PD-PRS, Long-Term TRAP Exposure, and Interaction and Joint Effects

Based on fully adjusted multivariable models, the marginal effects of the PD-PRS with PD showed that, as expected, each SD increase in the PRS was associated with an increased PD risk in both PEG (OR, 1.69; 95% CI, 1.49-1.91) and PASIDA (OR, 1.81; 95% CI, 1.64-2.00) (Table 2). The meta-analysis estimated an overall OR of 1.76 (95% CI, 1.63-1.90). For long-term TRAP exposure with a 5-year lag, each IQR increase in exposure was associated with an increase in PD risk in PEG (OR, 1.10; 95% CI, 1.04-1.16) and in PASIDA (OR, 1.09; 95% CI, 1.00-1.18). The meta-analysis also estimated an increase (OR, 1.10; 95% CI, 1.05-1.15).

**Table 2.** Marginal, Interaction, and Joint Effects of PD-PRS and Long-Term TRAP Exposure With a 5-Year Lag on PD Risk

Analysis	PEG study		PASIDA study		Meta-analysis	
	No. of cases/controls	OR (95% CI)^a^	No. of cases/controls	OR (95% CI)^b^	No. of cases/controls	OR (95% CI)
Marginal association						
PRS (per SD)	634/733	1.69 (1.49-1.91)	966/1045	1.81 (1.64-2.00)	1600/1778	1.76 (1.63-1.90)
TRAP (per IQR)	NA	1.10 (1.04-1.16)	NA	1.09 (1.00-1.18)	NA	1.10 (1.05-1.15)
Multiplicative interaction						
PRS (per SD) × TRAP (per IQR)	NA	1.07 (1.01-1.15)	NA	1.03 (0.94-1.14)	NA	1.06 (1.00-1.12)
Joint effect^c^						
Low PRS × low TRAP	318/460	1 [Reference]	470/652	1 [Reference]	788/1112	1 [Reference]
Low PRS × high TRAP	106/141	1.24 (0.90-1.70)	174/212	1.20 (0.94-1.53)	280/353	1.21 (1.00-1.47)
High PRS × low TRAP	145/102	2.15 (1.59-2.94)	244/142	2.42 (1.90-3.09)	389/244	2.31 (1.91-2.80)
High PRS × high TRAP	65/30	3.41 (2.13-5.57)	78/39	2.81 (1.86-4.29)	143/69	3.05 (2.23-4.19)
Expected joint effect	NA	2.67 (1.72-4.16)	NA	2.91 (2.06-4.10)	NA	2.80 (2.13-3.67)

Abbreviations: NA, not applicable; PASIDA, Parkinson Disease in Denmark; PD, Parkinson disease; PEG, Parkinson Environment and Genes; OR, odds ratio; PRS, polygenic risk score; TRAP, traffic-related air pollution.

^a^ Adjusted for age, gender, years of education, study wave, pesticide exposure, smoking status, family history of PD, job history, and 3 principal components of genetic ancestry.

^b^ Adjusted for age, gender, educational level, smoking status, family history of PD, job history, and 3 principal components of genetic ancestry.

^c^ Low (quartiles 1-3) and high (quartile 4) refer to quartiles of PRS and TRAP exposure.

Based on continuous variables, statistically significant multiplicative interactions between the PD-PRS and TRAP exposure were found in PEG (OR, 1.07; 95% CI, 1.01-1.15) but not in PASIDA (OR, 1.03; 95% CI, 0.94-1.14), with a pooled OR of 1.06 (95% CI, 1.00-1.12). When examining joint effects using dichotomous variables for the PRS and TRAP exposure (low: quartiles 1-3; high: quartile 4), the highest PD risk was estimated for both high PRS and high TRAP exposure compared with the reference group of low PRS and low TRAP exposure. In PEG, high PRS and high TRAP exposure was associated with an increased PD risk (OR, 3.41; 95% CI, 2.13-5.57), which was 28% higher than the expected joint effect (OR, 2.67; 95% CI, 1.72-4.16). In PASIDA, high PRS and high TRAP exposure nearly tripled the risk (OR, 2.81; 95% CI, 1.86-4.29). The meta-analytical joint effect (OR, 3.05; 95% CI, 2.23-4.19) was 9% higher than the expected joint effect (OR, 2.80; 95% CI, 2.13-3.67).

To explore potential risk gradients for genetic susceptibility interactions with TRAP, we categorized the PD-PRS into 3 levels (low: quartile 1; intermediate: quartiles 2-3; high: quartile 4) and examined the joint effects with binary TRAP exposure (low: quartiles 1-3; high: quartile 4). The risk of PD increased with both higher PRS and TRAP exposure, most strongly in PEG and the meta-analysis (Figure). While the PRS was the primary driver of PD risk, higher TRAP exposure showed a modest increased risk, particularly in the highest PRS category, despite overlapping CIs. Results from sensitivity analyses were robust, with positive multiplicative interactions consistently observed and strong joint effects for high PRS and high TRAP exposure (eTables 3-8 in Supplement 1). PEG and PASIDA results showed no evidence of nonlinearity (eFigure 5 in Supplement 1).

**Figure.**
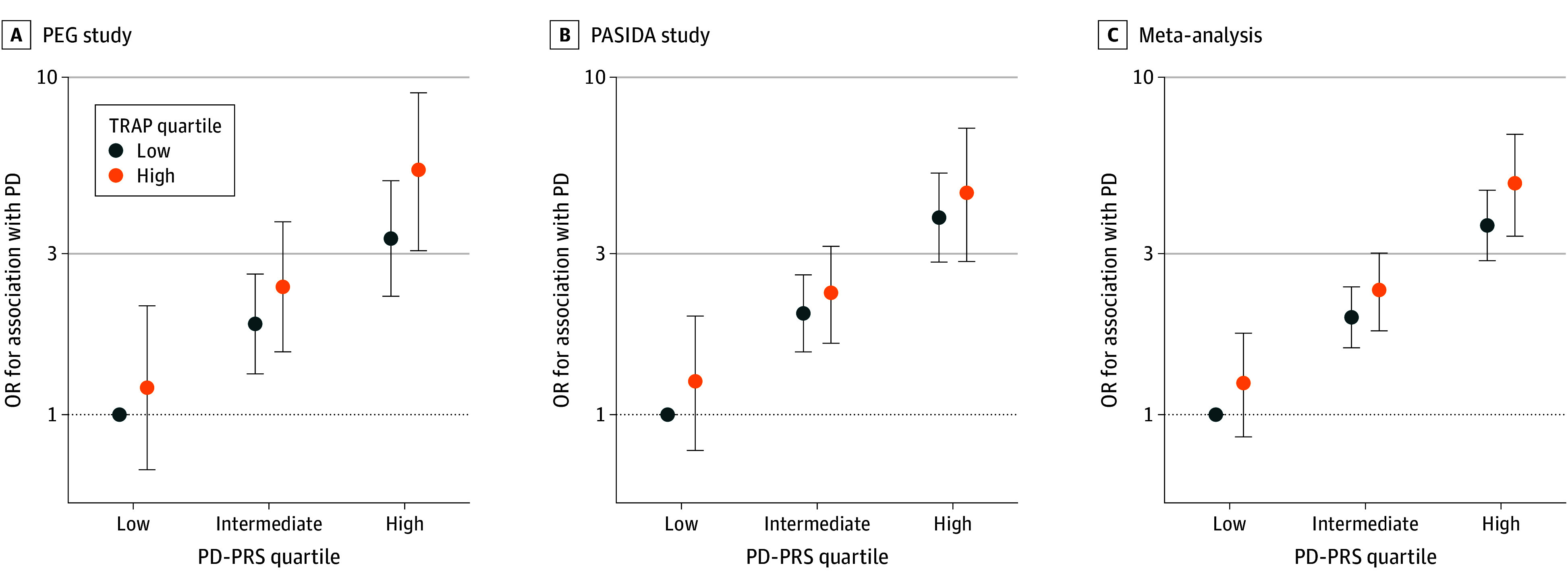
Joint Effects of Parkinson Disease–Polygenic Risk Score (PD-PRS) Quartile and Long-Term Traffic-Related Air Pollution (TRAP) Exposure Quartile PD-PRS quartiles are classified as low for quartile 1, intermediate for quartiles 2 and 3, and high for quartile 4. TRAP quartiles are classified as low for quartiles 1 to 3 and high for quartile 4. A, Analysis of the Parkinson Environment and Genes (PEG) study adjusted for age, gender, years of education, study wave, pesticide exposure, and 3 principal components (PCs) of genetic ancestry. B, Analysis of the Parkinson Disease in Denmark (PASIDA) study adjusted for age, gender, educational level, and 3 PCs of genetic ancestry. C, Meta-analysis included pooled estimates from the PEG and PASIDA studies. OR indicates odds ratio.

## Discussion

Our findings indicate gene-environment interactions between genetic susceptibility to PD and long-term TRAP exposure in PD, replicated in 2 large, independent, population-based case-control studies, PEG and PASIDA. Both studies incorporated more than 10 years of cumulative air pollution data (modeled CO levels as proxies for TRAP) in relevant exposure periods and genetic susceptibility (measured by PRS), showing independent associations of PRS and TRAP with PD risk, aligning with previous studies.^16,17^ Notably, joint effects of both risk factors were much more pronounced, with PD risk increasing up to 3-fold in genetically susceptible individuals exposed to high TRAP levels. Our findings suggest a synergistic effect, with the observed risk increase being larger than expected if both risk factors would act independently.

Mechanistically, TRAP likely triggers neuroinflammation and neurotoxic effects leading to PD. For example, diesel exhaust—a component of traffic pollution—was shown to induce selective neurotoxic effects in dopaminergic neurons, activate microglia in mesencephalic cultures,^32,33^ and increase α-synuclein levels (a hallmark protein for PD) in the midbrain.^32,34,35^ Polycyclic aromatic hydrocarbons, which are abundant in diesel exhaust, can easily enter the brain^36^ and facilitate neurotoxic effects, as seen in zebrafish.^37,38^ Additionally, evidence of increased inflammatory gene expression in the olfactory bulb of mice^34,35,39^ and neuroinflammation and α-synuclein accumulation observed in the brains of children and young adults who were chronically exposed to high levels of urban air pollution before death^40^ further suggest a role for TRAP in PD development, possibly through both neurotox effects and brain inflammation.

A prior study using the UK Biobank data (2356 patients with PD and 309 653 controls) also explored interactions between a PD-PRS and air pollution in PD^41^; we replicated those findings with a similar size joint effect. The UK Biobank study estimated statistically significant multiplicative interactions (*P* < .05) and strong joint effects for high exposure to several air pollutants (quartile 4) and a high PRS (tertile 3) and PD (hazard ratio [HR] for particulate matter <10 μm, 2.75 [95% CI, 2.15-3.52]; HR for NO_2_, 2.41 [95% CI, 1.91-3.05]; HR for NO_x_, 2.29 [95% CI, 1.82-2.89]) compared with low exposure (quartile 1) and low genetic risk (tertile 1). However, that study relied on codes from the *International Classification of Diseases, Ninth Revision* and *International Statistical Classification of Diseases, Tenth Revision* that included other parkinsonian disorders, such as secondary parkinsonism (eg, code G21); incorporated nonverified self-reported diagnoses; and examined shorter-term exposures (1-3 years).^41^ In contrast, our studies confirmed primary PD diagnoses through examinations by movement disorder specialists and record reviews and used longer-term exposure (≥10 years with a 5-year lag) to account for PD’s prodromal phase. Testing for PRS interactions with environmental factors requires large sample sizes to achieve sufficient statistical power, and environmental exposures have to be well measured, conditions that our studies satisfied. Additionally, we were able to replicate the findings from central California (PEG) in Denmark (PASIDA).

### Limitations

Our research has limitations, including potential exposure misclassification. The model-based estimates of TRAP did not account for personal behaviors or time spent in different environments, such as workplaces or during commutes. However, this misclassification is nondifferential, likely biasing effect estimates toward the null. Furthermore, our study focuses on outdoor air pollution (specifically TRAP), which does not reflect indoor pollution but is more relevant for regulatory actions. For PEG, we used CALINE4, which models TRAP only within 1.5 km of an address, while the PASIDA study’s TRAP model included local, area-wide, and regional sources. Therefore, direct comparisons of TRAP levels between central California and Denmark are not meaningful due to differences in the modeling approaches. However, we can assume that the relative ranking of exposure across study participants is valid, allowing effect estimates to be combined using meta-analytical approaches. The generalizability of our findings is limited to individuals of European ancestry, as the PRS was based on European populations only.

## Conclusions

In this case-control study of gene-environment interactions, we found that long-term TRAP exposure was particularly harmful to individuals genetically susceptible to PD, although TRAP negatively impacts the brains of older adults in general. While genetic susceptibility was a relevant PD risk factor for a subset of the population, the widespread exposure to air pollution makes TRAP an important modifiable risk factor affecting large populations globally. Future research is needed to replicate these results and investigate the biological mechanisms explaining these statistical interactions.

## Notes

Supplement 1. **eMethods.** Participants and Procedures **eFigure 1.** PEG Study Population Flowchart **eFigure 2.** PASIDA Study Population Flowchart **eFigure 3.** DAG Illustrating the Association Among PD-PRS, TRAP Exposure, and PD **eFigure 4.** Scatterplot of PD-PRS and Long-Term TRAP Exposure With a 5-Year Lag Time **eFigure 5.** Nonlinear Associations Between 10-Year Mean CO Exposure per IQR Increase and PD Risk in the PEG and PASIDA Studies **eTable 1.** Descriptive Statistics for Annual TRAP Exposures at Residential Addresses **eTable 2.** Heterogeneity Metrics From Meta-analyses Conducted for Fully Adjusted Models Across the PEG and PASIDA Studies **eTable 3.** Crude Model of PD Risk: Marginal, Interaction, and Joint Effects of PD-PRS and Long-Term TRAP With a 5-Year Lag **eTable 4.** PD Risk With Alternate PD-PRS Using 76 SNVs: Marginal, Interaction, and Joint Effects **eTable 5.** PD Risk With Complete Residential Address History: Marginal, Interaction, and Joint Effects **eTable 6.** PD Risk With PEG Adjusting for Neighborhood SES: Marginal, Interaction, and Joint Effects **eTable 7.** PD Risk With PASIDA 10-Year Exposure Period With a 5-Year Lag: Marginal, Interaction, and Joint Effects **eTable 8.** PD Risk Including Only PASIDA Incident Cases Diagnosed Between 2006 and 2009: Marginal, Interaction, and Joint Effects
eReferences.

Supplement 2.
Data Sharing Statement
