# Supplementary material for: Interaction Between Traffic-Related Air Pollution and Parkinson Disease Polygenic Risk Score
Source: JAMA Netw Open. 2025 Mar 17;8(3):e250854. doi: 10.1001/jamanetworkopen.2025.0854 (PMC11915066; doi:10.1001/jamanetworkopen.2025.0854)
Supplement: Supplement 1. — eMethods. Participants and Procedures eFigure 1. PEG Study Population Flowchart eFigure 2. PASIDA Study Population Flowchart eFigure 3. DAG Illustrating the Association Among PD-PRS, TRAP Exposure, and PD eFigure 4. Scatterplot of PD-PRS and Long-Term TRAP Exposure With a 5-Year Lag Time eFigure 5. Nonlinear Associations Between 10-Year Mean CO Exposure per IQR Increase and PD Risk in the PEG and PASIDA Studies eTable 1. Descriptive Statistics for Annual TRAP Exposures at Residential Addresses eTable 2. Heterogeneity Metrics From Meta-analyses Conducted for Fully Adjusted Models Across the PEG and PASIDA Studies eTable 3. Crude Model of PD Risk: Marginal, Interaction, and Joint Effects of PD-PRS and Long-Term TRAP With a 5-Year Lag eTable 4. PD Risk With Alternate PD-PRS Using 76 SNVs: Marginal, Interaction, and Joint Effects eTable 5. PD Risk With Complete Residential Address History: Marginal, Interaction, and Joint Effects eTable 6. PD Risk With PEG Adjusting for Neighborhood SES: Marginal, Interaction, and Joint Effects eTable 7. PD Risk With PASIDA 10-Year Exposure Period With a 5-Year Lag: Marginal, Interaction, and Joint Effects eTable 8. PD Risk Including Only PASIDA Incident Cases Diagnosed Between 2006 and 2009: Marginal, Interaction, and Joint Effects eReferences. [file jamanetwopen-e250854-s001.pdf]

Supplemental Online Content

Kwon D, Paul KC, Kusters C, et al. Interaction between traffic-related air pollution and Parkinson disease polygenic risk score. 2025;8(3):e250854.
doi:10.1001/jamanetworkopen.2025.0854

eMethods.
eFigure 1. PEG Study Population Flowchart
eFigure 2. PASIDA Study Population Flowchart
eFigure 3. DAG Illustrating the Association Among PD-PRS, TRAP Exposure, and PD
eFigure 4. Scatterplot of PD-PRS and Long-Term TRAP Exposure with a 5-Year Lag Time
eFigure 5. Nonlinear Associations Between 10-Year Mean CO Exposure per IQR Increase and PD Risk in the PEG and PASIDA Studies
eTable 1. Descriptive Statistics for Annual TRAP Exposures at Residential Addresses
eTable 2. Heterogeneity Metrics from Meta-Analyses Conducted for Fully Adjusted Models Across the PEG and PASIDA Studies
eTable 3. Crude Model of PD Risk: Marginal, Interaction, and Joint Effects of PD-PRS and Long-Term TRAP with a 5-Year Lag
eTable 4. PD Risk with Alternate PD-PRS Using 76 SNVs: Marginal, Interaction, and Joint Effects
eTable 5. PD Risk with Complete Residential Address History: Marginal, Interaction, and Joint Effects
eTable 6. PD Risk with PEG Adjusting for Neighborhood SES: Marginal, Interaction, and Joint Effects
eTable 7. PD Risk with PASIDA 10-Year Exposure Period With a 5-Year Lag: Marginal, Interaction, and Joint Effects
eTable 8. PD Risk Including Only PASIDA Incident Cases Diagnosed Between 2006 and 2009: Marginal, Interaction, and Joint Effects
eReferences

This supplemental material has been provided by the authors to give readers additional information about their work.

© 2025 Kwon D et al. *JAMA Network Open*.

eMethods

Study population

The PEG study ensured minimal missingness by conducting extensive in-person physical assessments and structured interviews with participants, during which key demographic, lifetime residential address history, and medical history data were consistently collected. Similarly, the PASIDA study leveraged Denmark's comprehensive national registries, and we also conducted detailed thorough medical history reviews and in-person interviews, further reducing the chance of missing data. As a result of using multiple data sources to ascertain the key variables, there was no missingness in covariates used in our analysis for either study.

Air pollution exposure assessment

The CALINE4[1,2] dispersion model was used in the PEG study to estimate annual TRAP levels, represented by CO, within a 1.5 km buffer zone around each participant's residential address, starting from 1981. This buffer was chosen to model local traffic emissions accurately, capturing relevant meteorological conditions and roadway geometry within the zone. While CO levels drop quickly with distance from main roads due to dilution and mixing, the 1.5 km buffer reflects the CALINE4 model's design to capture the most relevant distance for average yearly TRAP exposure, rather than being a study-specific decision. Additionally, nighttime concentrations of ultrafine particles can extend as far as 2 km, while daytime levels may return to background concentrations within 300 m. Emissions from sources beyond the buffer, such as regional pollution or non-traffic sources, were not included, as the CALINE4 model is focused on local sources.

In contrast, CO in PASIDA represents a combination of local, area-based, and regional pollution, modeled by the Danish DEHM/UBM/AirGIS dispersion model, with data available from 1971. Thus, while absolute CO levels are not directly comparable between the two studies, the relative ranking of exposure across study participants remains valid for meta-analysis.

In both the PEG and PASIDA studies, CO was selected as the primary marker of traffic exhaust instead of NO_2. Historically, CO was a reliable TRAP marker, especially from the 1980s through the early 2000s[3,4] – the exposure period relevant to these studies. During this time, vehicle emissions were higher due to less efficient combustion and limited emission control technologies.[3,4] Unlike NO_2, CO does not undergo atmospheric chemical reactions, which makes it a more direct representation of tailpipe emissions. NO_2 from traffic-related combustion reacts with other chemicals in the atmosphere, forming particulate matter and ozone, while CO remains stable and thus better represents direct traffic exhaust.[4] Additionally, regulatory agencies prioritized CO due to its potential immediate effects on health, leading to widespread monitoring networks.[4] However, as vehicle technology advanced and regulations such as the US Clean Air Act took effect, CO levels dropped considerably,[3] making pollutants like NO and NO_2 the common TRAP markers in recent years, beyond our study period.

To account for variability in TRAP exposure across participants, effect estimates were reported per IQR increase in CO exposure, a standard approach in air pollution studies. This allows for comparability across studies and ensures that our interpretation of results based on different exposure distributions and sources in each study is more robust.

Pesticide exposure assessment in PEG

The PEG study was conducted in central California, an agriculturally rich region where pesticide use is common. This geographic characteristic makes pesticide exposure an important environmental factor for consideration, as previous studies have demonstrated that pesticide exposure is associated with increased risk and progression of PD.[5,6] Of the 634 PD cases in PEG, 514 (81%) were exposed to pesticides during the exposure period of interest, while 542 (74%) of the 734 controls had similar pesticide exposure within the 500 m buffer zone.

We assessed exposure to specific pesticide active ingredients near agricultural pesticide application sites using recorded data and a geographic information system-based model.[7] Since 1974, California law has required the recording of all commercial agricultural pesticide use. This information was stored in the Pesticide Use Reporting database of the California Department of Pesticide Regulation, which provides detailed records of application

© 2025 Kwon D et al. *JAMA Network Open*.

locations, poundage, crop type, acreage, and application methods. This comprehensive database spans over 40 years of agricultural pesticide applications in the tri-county area (Fresno, Kern, and Tulare) during 1974-2017.

Using lang-use and crop cover maps, we identified pesticide applications at specific agricultural sites.[8] Our participants provided lifetime residential addresses, which were geocoded using a multi-step process.[9] For each pesticide and participant, we calculated pounds of pesticide applied per acre within a 500 m buffer around each address annually since 1974. The total poundage of pesticide applied was then weighted by the proportion of acreage treated (lbs/acre), allowing us to estimate each participant's pesticide exposure at their residential location.[6]

In contrast, equivalent pesticide data were not available for the PASIDA study, and pesticide exposure was not considered relevant due to differences in agricultural practices. Specifically, 80% of PASIDA participants lived in urban or provincial city settings in Denmark, where agricultural pesticide use is far less prevalent and largely restricted to greenhouse applications. As a result, the general public's exposure to pesticides in Denmark is lower than in California.

Genotyping, Quality Control and calculation of the PRS

Genotyping was performed using the Global Screening Array (GSA v1, Illumina, Inc) as previously described.[10,11] Genotyping was conducted at the UKSH NGS facility at the Institute for Clinical Molecular Biology (IKMB) in Kiel, Germany. Post-genotyping data processing and imputation took place at the UKSH campus in Lübeck and the University of Lübeck. Raw data processing, including clustering and genotype calling from raw intensity data, was performed using GenomeStudio v2.0.4 with the "GSAsharedCUSTOM_20018389_A6" manifest (Illumina, Inc.), which annotates 696,375 variants. Variants with GenTrain values below 0.7, samples with a call rate under 95%, and samples with a GenCall Score below 0.7 were excluded at this stage.

Further quality control (QC) filtering, including sex checks, strand checks, and flipping, was conducted using PLINK v1.9. SNVs with a missing genotype rate over 2% and subjects with a genotyping call rate under 95% were removed. Additionally, SNVs failing the Hardy-Weinberg equilibrium (HWE) test with a p-value less than 5e-6, and variants with a minor allele frequency (MAF) below 1% were excluded. To identify cryptic relatedness, pairwise allele sharing was determined using a linkage disequilibrium (LD)-pruned set of markers and pairwise allele-sharing identity IBD/IBS. This process resulted in 495,338 QC-filtered SNVs across 1,866 PEG and 3,486 PASIDA samples suitable for imputation. The LD-pruned dataset was also utilized for principal component analysis, along with the 1000 Genome Project Consortium Phase 3 reference dataset (The 1000 Genomes Project Consortium, 2015). Ethnic descent groups were assigned using the five 1000G super-populations by k-nearest neighbor classification with the 'class' package in R 2.3.2.

Before imputation, the quality-controlled genotype data were subjected to bcftools (v1.9) and QC'ed again (removal of ambiguous SNVs, re-checking, and correcting strand mismatches). This was followed by haplotype phasing using SHAPEIT2 and imputation of unobserved genotypes using Minimac3, based on a precompiled Haplotype Reference Consortium (HRC) reference panel (EGAD00001002729, including 39,131,578 SNVs from ~11K individuals).

After imputation, we retained bi-allelic autosomal SNVs with a minimac3 $R^2 \geq 0.3$. Genotyped variants were hard-called using an 80% probability imputation. A total of 38,913,048 bi-allelic variants remained among both PEG and PASIDA. We excluded variants with a MAF less than 1% (n = 31,179,961 for PEG; n = 31,388,057 for PASIDA), variants with a HWE test p-value $<1x10^{-7}$ (n = 53,327 for PEG; n = 342 for PASIDA), and variants with a call rate <90% (n = 309,278 for PEG; n = 218,493 for PASIDA), leaving 7,370,482 variants among PEG and 7,306,156 variants among PASIDA participants.

Of the 90 SNVs identified in Nalls et al,[12] two SNVs were not available in both studies (rs34637584 – LRRK2; rs76763715 – GBAP1), and two additional SNVs were not available in one of the studies (rs114138760 - PMVK in PEG; rs55818311 – SPPL2B in PASIDA), so these were excluded from the PRS to ensure comparability between the two studies. This resulted in 86 SNVs that were available in both the PEG and PASIDA datasets.

After identifying the SNVs for the PRS, we aligned the effect alleles between Nalls et al.[12] and our datasets. For each individual, we multiplied the effect estimate (betas) by the number of effect alleles for each SNV and then

© 2025 Kwon D et al. *JAMA Network Open*.

summed these values to obtain a single PRS. Finally, the PRS was standardized and centered within each study to assess the effects of the PRS per SD, as common in genetic studies. This allows for appropriate scaling and interpretation of genetic risk.

As a sensitivity analysis, and to account for the potential overestimation of genetic risk in a PRS (due to LD between SNVs), we also created a PRS with a stringent clumping threshold to ensure independence between genetic variants. We used an LD threshold of $r^2 = 0.001$ and a 1 MB base pair window, based on the European reference within the 'TwoSampleMR' package in R. This restricted the PRS to 76 SNVs (correlation between the original and restricted PRS is 0.95).

SES adjustment and confounding

Education was used as a proxy for individual-level SES (iSES), effectively capturing much of the variation relevant to TRAP exposure. As our primary interest lies in GxE interactions and PD risk, the minimal sufficient adjustment set used in our analysis—including age, sex, genetic ancestry, education, pesticide exposure (for PEG), smoking status, family history of PD, job history, and study wave (for PEG)—likely indirectly adjusts for neighborhood-level SES (nSES) through the inclusion of education (eFigure 3). This adjustment set was designed to close relevant backdoor (confounding) paths between SES and PD.

Furthermore, the relationship between nSES and TRAP exposure differs across settings. In urban areas in the US, low nSES often correlates with higher air pollution exposure. However, in the PEG study conducted in California's rural Central Valley, this pattern may not hold (eTable 6), as the most deprived populations are often found in more marginalized areas. In PASIDA, a Danish study found no association between nSES and TRAP exposure.[13] In Denmark, wealthier individuals often live in inner-city areas with higher pollution, contrasting with urban settings in the US.

For residual confounding to meaningfully bias our results, nSES would need to be strongly associated with both TRAP exposure and PD risk. However, the unique socio-geographic patterns in both study settings make this unlikely. Thus, while we acknowledge the potential for residual confounding, its expected impact on our findings is minimal, with no evidence suggesting bias in the observed associations.

Meta-analyses and heterogeneity assessment

Heterogeneity was assessed using Cochran's Q test and I^2 statistics. For interaction term in meta-analyses, heterogeneity was not detected (Q = 0.41, p-value = 0.52; I^2 = 0.00%; eTable 2), suggesting that the interaction effects observed across locations were similar enough to justify pooling the results. This lack of heterogeneity may reflect the harmonized data collection and analysis methods across the two studies, including consistent exposure assessing that used CO as a marker for TRAP, application of a 5-year lag period, and adjustment for a similar set of covariates. However, the possibility of limited statistical power to detect heterogeneity given the inclusion of only two studies cannot be ruled out.

Although the point estimates for interaction effects in PEG and PASIDA showed some differences (Table 2), these differences were within the range expected from random variation. The pooled interaction OR of 1.06 (95% CI = 1.00-1.12) reflects the overall trend across the studies. The decision to pool these results was supported by the lack of heterogeneity. Small differences in point estimates do not necessarily indicate meaningful differences in interaction effects, and pooling the results provides a more comprehensive assessment of the interaction effects across the two locations.

© 2025 Kwon D et al. *JAMA Network Open*.

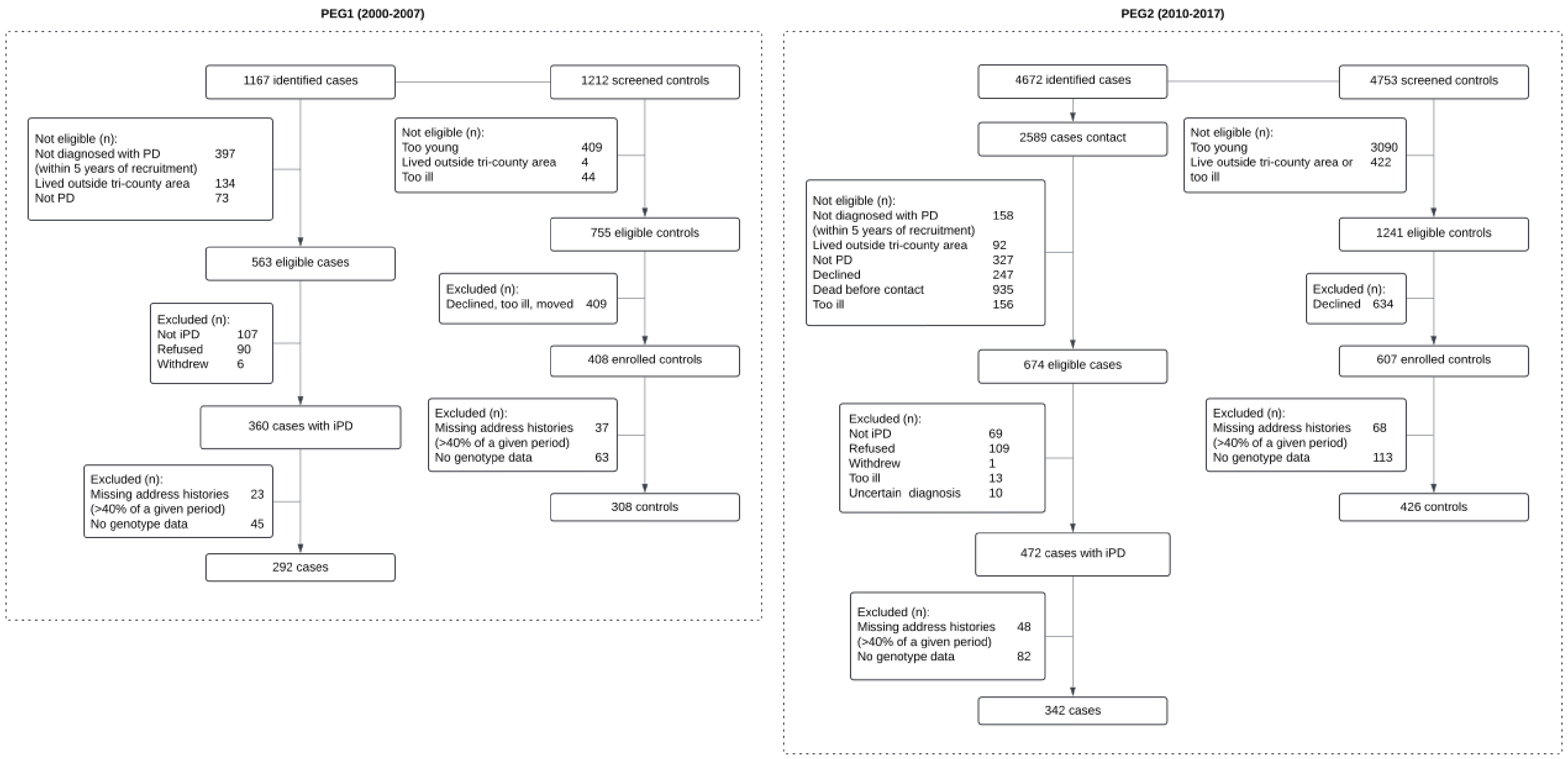

eFigure 1. PEG Study Population Flowchart

© 2025 Kwon D et al. *JAMA Network Open*.

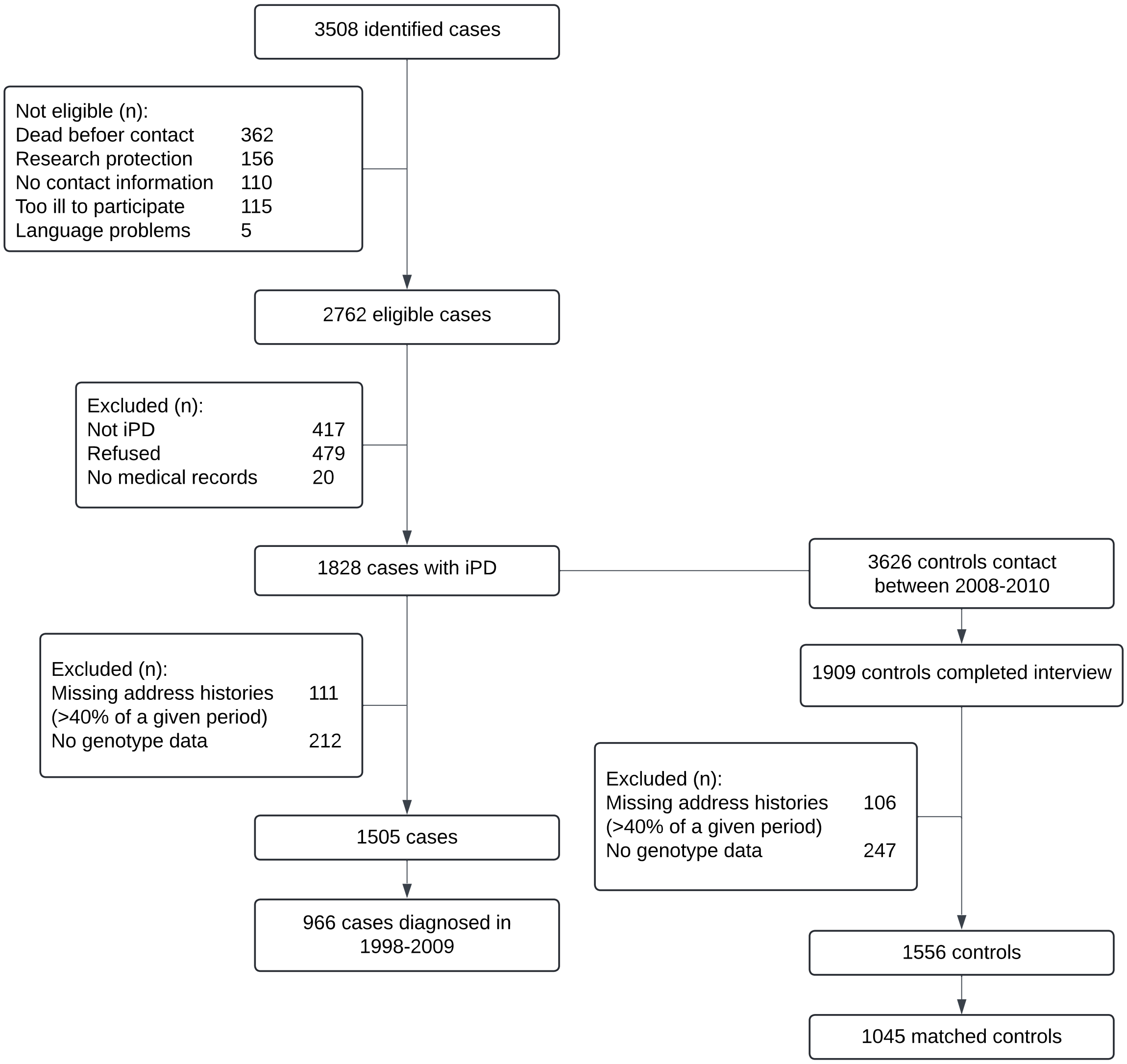

eFigure 2. PASIDA Study Population Flowchart

© 2025 Kwon D et al. *JAMA Network Open*.

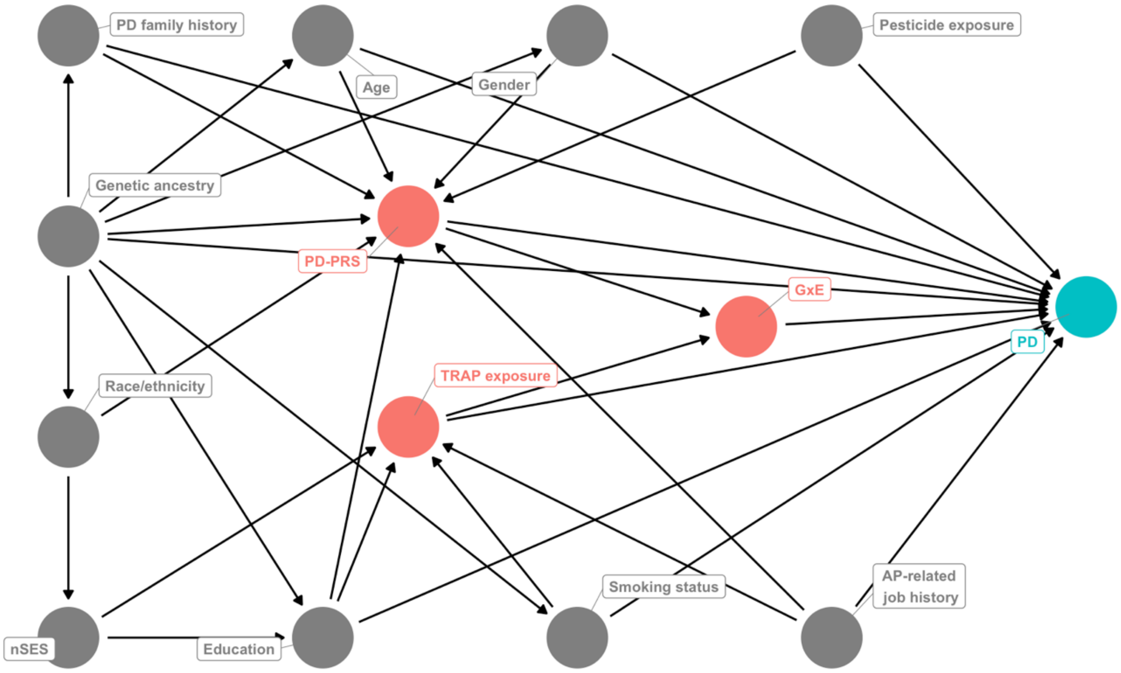

eFigure 3. DAG Illustrating the Association Among PD-PRS, TRAP Exposure, and PD. Abbreviations: DAG, directed acyclic graph; PD, Parkinson disease; PRS, polygenic risk score; TRAP, traffic-related air pollution; GxE, gene-environment interaction; nSES, neighborhood-level socioeconomic status.

© 2025 Kwon D et al. *JAMA Network Open*.

a. PEG study

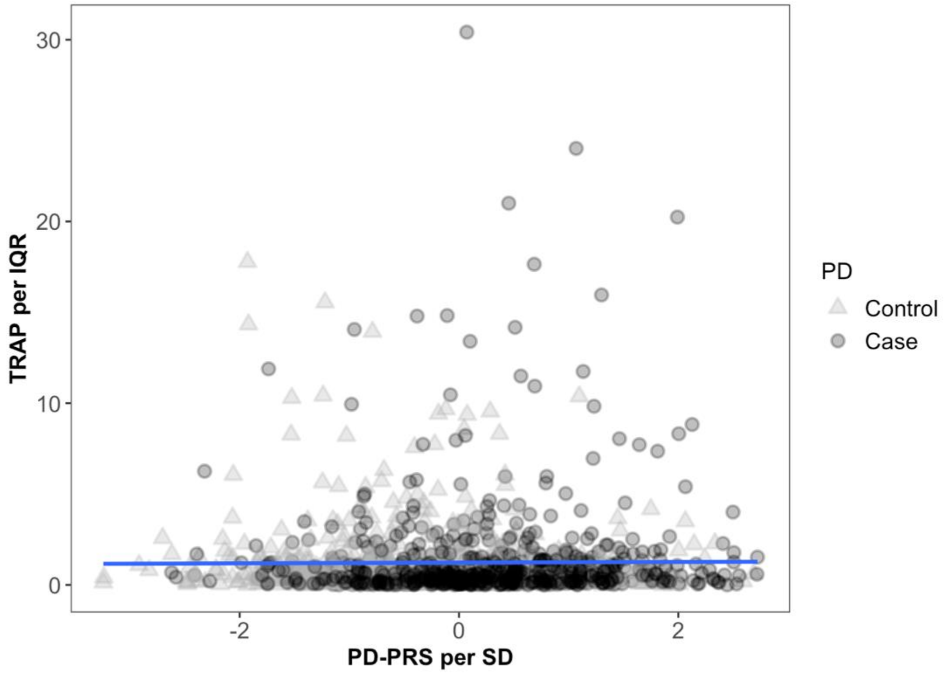

b. PASIDA study

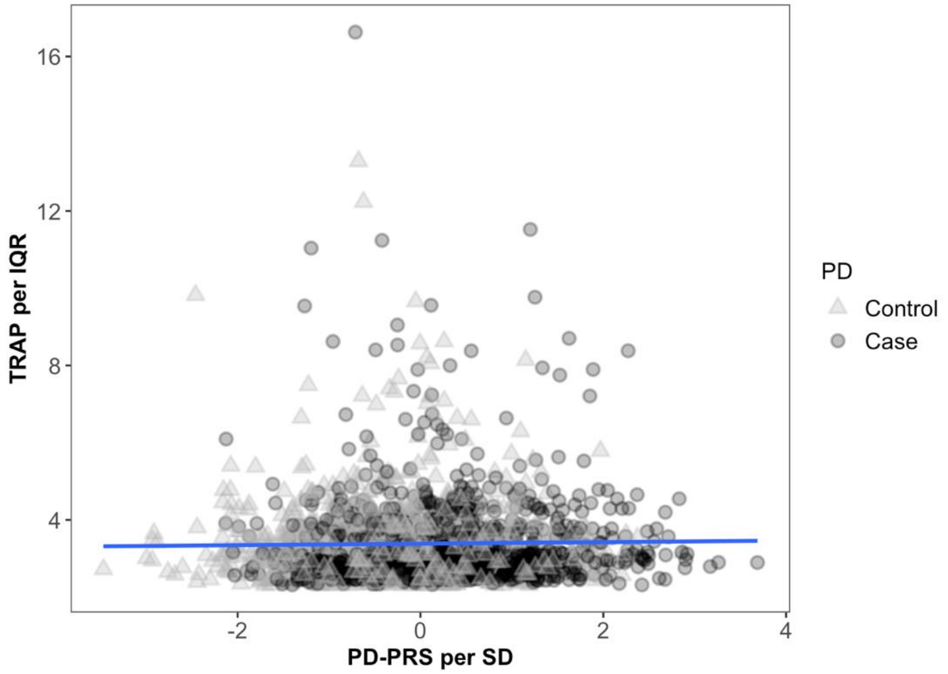

eFigure 4. Scatterplot of PD-PRS and Long-Term TRAP Exposure with a 5-Year Lag Time. **a.** PEG study: gene-environment independence in total study population (Spearman's rank correlation rho = 0.02, *p* = 0.38) and controls (rho = -0.06, *p* = 0.13). **b.** PASIDA study: gene-environment independence in total study population (rho = 0.03, *p* = 0.19) and controls (rho = 0.009, *p* = 0.78). Abbreviations: PD, Parkinson disease; PRS, polygenic risk score; TRAP, traffic-related air pollution; SD, standard deviation; IQR, interquartile range.

© 2025 Kwon D et al. *JAMA Network Open*.

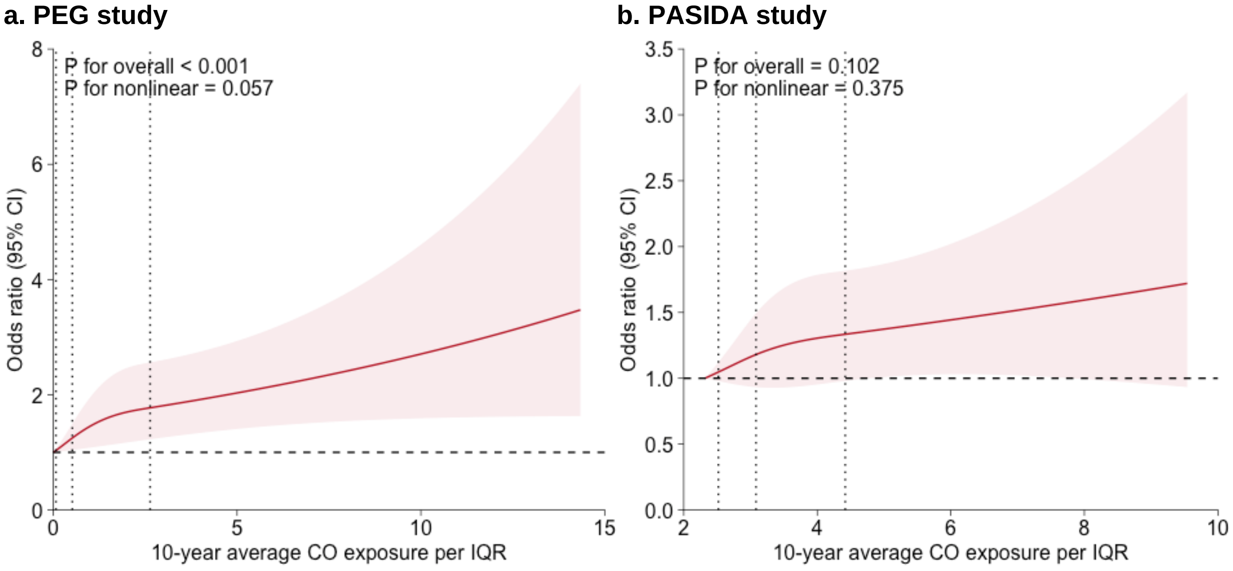

eFigure 5. Nonlinear Associations Between 10-Year Mean CO Exposure per IQR Increase and PD Risk in the PEG and PASIDA Studies. Restricted cubic spline models with three knots (10th, 50th, and 90th percentiles of CO exposure) were used. Models for PEG were adjusted for age, gender, years of education, study wave, pesticide exposure, smoking status, family history of PD, job history, and 3 PCs of genetic ancestry. Models for PASIDA were adjusted for age, gender, education level, smoking status, family history of PD, job history, and 3 PCs of genetic ancestry. Abbreviations: CO, carbon monoxide; IQR, interquartile range; PD, Parkinson disease; CI, confidence interval.

© 2025 Kwon D et al. *JAMA Network Open*.

eTable 1. Descriptive Statistics for Annual TRAP Exposures at Residential Addresses[a]

			Percentile						Pearson correlation		
	Mean ± SD	IQR	Min	25th	50th	75th	95th	Max	CO	NO_2	NO_x
**PEG** (Period: 1981-2016)											
CO (ppb)	12.33 ± 29.89	10.12	0.00	0.91	3.44	11.02	51.47	671.87			
**PASIDA** (Period: 1971-2009)											
CO (ppm)	0.54 ± 0.34	0.45	0.16	0.27	0.49	0.72	1.04	6.86	1		
NO_2 ($\mu g/m^3$)	13.74 ± 5.26	3.50	3.76	11.07	12.28	15.47	23.56	66.54	0.82	1	
NO_x ($\mu g/m^3$)	21.09 ± 18.37	5.55	4.88	14.15	16.02	19.70	47.71	334.51	0.84	0.93	1

Abbreviations: TRAP, traffic-related air pollution; NO_2, nitrogen dioxide; NO_x, nitrogen oxide; CO, carbon monoxide; SD, standard deviation; IQR, interquartile range.

[a]TRAP indicators are based on model-based estimates of air pollutant levels, not direct measurements. The PEG study used local traffic data, while the PASIDA study combined local and regional data.

© 2025 Kwon D et al. *JAMA Network Open*.

eTable 2. Heterogeneity Metrics from Meta-analyses Conducted for Fully Adjusted Models Across the PEG and PASIDA Studies

	Q	P value	I^2
Marginal effect			
PRS (per SD)	0.72	0.40	0.00
TRAP (per IQR)	0.03	0.86	0.00
Multiplicative interaction			
PRS (per SD) x TRAP (per IQR)	0.41	0.52	0.00
Joint effect			
Low PRS x Low TRAP			
Low PRS x High TRAP	0.03	0.87	0.00
High PRS x Low TRAP	0.35	0.55	0.00
High PRS x High TRAP	0.35	0.55	0.00

Abbreviations: PRS, polygenic risk score; TRAP, traffic-related air pollution; SD, standard deviation; IQR, interquartile range.

© 2025 Kwon D et al. *JAMA Network Open*.

eTable 3. Crude Model of PD Risk: Marginal, Interaction, and Joint Effects of PD-PRS and Long-Term TRAP with a 5-Year Lag

	**PEG study**		**PASIDA study**		**Meta-analysis**	
	Case/control	OR (95% CI)	Case/control	OR (95% CI)	Case/control	OR (95% CI)
Marginal effect						
PRS (per SD)	634/733	1.64 (1.46-1.84)	966/1045	1.80 (1.63-1.99)	1600/1778	1.73 (1.60-1.87)
TRAP (per IQR)		1.07 (1.02-1.13)		1.07 (0.99-1.16)		1.07 (1.02-1.12)
Multiplicative interaction						
PRS (per SD) x TRAP (per IQR)		1.07 (1.01-1.15)		1.03 (0.94-1.13)		1.06 (1.00-1.11)
Joint effect[a]						
Low PRS x Low TRAP	318/460	1.00	470/652	1.00	788/1112	1.00
Low PRS x High TRAP	106/141	1.09 (0.81-1.45)	174/212	1.14 (0.90-1.44)	280/353	1.12 (0.93-1.34)
High PRS x Low TRAP	145/102	2.06 (1.54-2.76)	244/142	2.38 (1.88-3.03)	389/244	2.25 (1.87-2.70)
High PRS x High TRAP	65/30	3.13 (2.01-5.00)	78/39	2.77 (1.87-4.18)	143/69	2.92 (2.16-3.95)
Expected joint effect		2.24 (1.48-3.37)		2.71 (1.94-3.79)		2.52 (1.94-3.26)

Abbreviations: PD, Parkinson disease; PRS, polygenic risk score; TRAP, traffic-related air pollution; SD, standard deviation; IQR, interquartile range.
[a]Low (Q1-3) and high (Q4) refer to quartiles of PRS and TRAP exposure.

© 2025 Kwon D et al. *JAMA Network Open*.

eTable 4. PD Risk with Alternate PD-PRS Using 76 SNVs: Marginal, Interaction, and Joint Effects

	**PEG study**		**PASIDA study**		**Meta-analysis**	
	Case/control	OR (95% CI)[a]	Case/control	OR (95% CI)[b]	Case/control	OR (95% CI)
Marginal effect						
PRS (per SD)	634/733	1.66 (1.46-1.88)	966/1045	1.74 (1.58-1.92)	1600/1778	1.71 (1.58-1.85)
TRAP (per IQR)		1.10 (1.04-1.16)		1.09 (1.00-1.18)		1.10 (1.05-1.15)
Multiplicative interaction						
PRS (per SD) x TRAP (per IQR)		1.06 (1.00-1.14)		1.03 (0.94-1.15)		1.05 (0.99-1.11)
Joint effect[c]						
Low PRS x Low TRAP	318/460	1.00	470/652	1.00	788/1112	1.00
Low PRS x High TRAP	106/141	1.25 (0.91-1.72)	174/212	1.12 (0.88-1.43)	280/353	1.17 (0.96-1.41)
High PRS x Low TRAP	145/102	2.15 (1.58-2.93)	244/142	2.30 (1.81-2.94)	389/244	2.24 (1.85-2.71)
High PRS x High TRAP	65/30	3.33 (2.08-5.44)	78/39	3.56 (2.33-5.56)	143/69	3.45 (2.50-4.77)
Expected joint effect		2.69 (1.73-4.18)		2.58 (1.83-3.64)		2.62 (2.00-3.44)

Abbreviations: PD, Parkinson disease; PRS, polygenic risk score; TRAP, traffic-related air pollution; SD, standard deviation; IQR, interquartile range.
[a]Adjusted for age, gender, years of education, study wave, pesticide exposure, smoking status, family history of PD, job history, and 3 PCs of genetic ancestry.
[b]Adjusted for age, gender, education level, smoking status, family history of PD, job history, and 3 PCs of genetic ancestry.
[c]Low (Q1-3) and high (Q4) refer to quartiles of PRS and TRAP exposure.

© 2025 Kwon D et al. *JAMA Network Open*.

eTable 5. PD Risk with Complete Residential Address History: Marginal, Interaction, and Joint Effects

	**PEG study**		**PASIDA study**		**Meta-analysis**	
	Case/control	OR (95% CI)[a]	Case/control	OR (95% CI)[b]	Case/control	OR (95% CI)
Marginal effect						
PRS (per SD)	575/666	1.70 (1.49-1.94)	948/1025	1.82 (1.65-2.02)	1523/1691	1.77 (1.64-1.92)
TRAP (per IQR)		1.10 (1.04-1.17)		1.09 (1.00-1.18)		1.10 (1.05-1.15)
Multiplicative interaction						
PRS (per SD) x TRAP (per IQR)		1.07 (1.01-1.15)		1.03 (0.94-1.13)		1.06 (1.00-1.12)
Joint effect[c]						
Low PRS x Low TRAP	283/425	1.00	461/642	1.00	744/1067	1.00
Low PRS x High TRAP	99/124	1.34 (0.96-1.87)	169/208	1.21 (0.94-1.54)	268/332	1.25 (1.03-1.53)
High PRS x Low TRAP	132/91	2.27 (1.64-3.15)	241/136	2.51 (1.96-3.22)	373/227	2.42 (1.99-2.95)
High PRS x High TRAP	61/26	3.95 (2.40-6.66)	77/39	2.75 (1.82-4.21)	138/65	3.18 (2.30-4.40)
Expected joint effect		3.04 (1.91-4.85)		3.03 (2.14-4.29)		3.02 (2.29-4.00)

Abbreviations: PD, Parkinson disease; PRS, polygenic risk score; TRAP, traffic-related air pollution; SD, standard deviation; IQR, interquartile range; PC, principal component.

[a]Adjusted for age, gender, years of education, study wave, pesticide exposure, smoking status, family history of PD, job history, and 3 PCs of genetic ancestry.

[b]Adjusted for age, gender, education level, smoking status, family history of PD, job history, and 3 PCs of genetic ancestry.

[c]Low (Q1-3) and high (Q4) refer to quartiles of PRS and TRAP exposure.

© 2025 Kwon D et al. *JAMA Network Open*.

eTable 6. PD Risk with PEG Adjusting for Neighborhood SES: Marginal, Interaction, and Joint Effects

	**PEG study**		**PASIDA study**		**Meta-analysis**	
	Case/control	OR (95% CI)[a]	Case/control	OR (95% CI)[b]	Case/control	OR (95% CI)
Marginal effect						
PRS (per SD)	634/733	1.69 (1.49-1.92)	966/1045	1.81 (1.64-2.00)	1600/1778	1.76 (1.63-1.91)
TRAP (per IQR)		1.10 (1.04-1.16)		1.09 (1.00-1.18)		1.10 (1.05-1.15)
Multiplicative interaction						
PRS (per SD) x TRAP (per IQR)		1.07 (1.01-1.15)		1.03 (0.94-1.14)		1.06 (1.00-1.12)
Joint effect[c]						
Low PRS x Low TRAP	318/460	1.00	470/652	1.00	788/1112	1.00
Low PRS x High TRAP	106/141	1.24 (0.90-1.70)	174/212	1.20 (0.94-1.53)	280/353	1.21 (1.00-1.47)
High PRS x Low TRAP	145/102	2.16 (1.59-2.94)	244/142	2.42 (1.90-3.09)	389/244	2.32 (1.91-2.80)
High PRS x High TRAP	65/30	3.40 (2.12-5.56)	78/39	2.81 (1.86-4.29)	143/69	3.05 (2.22-4.18)
Expected joint effect		2.67 (1.71-4.16)		2.91 (2.06-4.10)		2.81 (2.14-3.68)

Abbreviations: PD, Parkinson disease; PRS, polygenic risk score; TRAP, traffic-related air pollution; SD, standard deviation; IQR, interquartile range; PC, principal component.
[a]Adjusted for age, gender, years of education, study wave, pesticide exposure, smoking status, family history of PD, job history, nSES, and 3 PCs of genetic ancestry.
[b]Adjusted for age, gender, education level, smoking status, family history of PD, job history, and 3 PCs of genetic ancestry.
[c]Low (Q1-3) and high (Q4) refer to quartiles of PRS and TRAP exposure.

© 2025 Kwon D et al. *JAMA Network Open*.

eTable 7. PD Risk with PASIDA 10-Year Exposure Period with a 5-Year Lag: Marginal, Interaction, and Joint Effects

	PEG study		PASIDA study		Meta-analysis	
	Case/control	OR (95% CI)[a]	Case/control	OR (95% CI)[b]	Case/control	OR (95% CI)
Marginal effect						
PRS (per SD)	634/733	1.69 (1.49-1.91)	966/1045	1.81 (1.64-2.00)	1600/1778	1.76 (1.63-1.90)
TRAP (per IQR)		1.10 (1.04-1.16)		1.07 (0.98-1.16)		1.09 (1.04-1.14)
Multiplicative interaction						
PRS (per SD) x TRAP (per IQR)		1.07 (1.01-1.15)		1.02 (0.93-1.13)		1.05 (1.00-1.11)
Joint effect[c]						
Low PRS x Low TRAP	318/460	1.00	471/655	1.00	789/1115	1.00
Low PRS x High TRAP	106/141	1.24 (0.90-1.70)	173/209	1.21 (0.95-1.54)	279/350	1.22 (1.01-1.48)
High PRS x Low TRAP	145/102	2.15 (1.59-2.94)	243/139	2.47 (1.94-3.16)	388/241	2.34 (1.93-2.83)
High PRS x High TRAP	65/30	3.41 (2.13-5.57)	79/42	2.60 (1.74-3.93)	144/72	2.91 (2.13-3.97)
Expected joint effect		2.67 (1.72-4.16)		2.98 (2.11-4.21)		2.85 (2.18-3.74)

Abbreviations: PD, Parkinson disease; PRS, polygenic risk score; TRAP, traffic-related air pollution; SD, standard deviation; IQR, interquartile range; PC, principal component.
[a]Adjusted for age, gender, years of education, study wave, pesticide exposure, smoking status, family history of PD, job history, and 3 PCs of genetic ancestry.
[b]Adjusted for age, gender, education level, smoking status, family history of PD, job history, and 3 PCs of genetic ancestry.
[c]Low (Q1-3) and high (Q4) refer to quartiles of PRS and TRAP exposure.

© 2025 Kwon D et al. *JAMA Network Open*.

eTable 8. PD Risk Including Only PASIDA Incident Cases Diagnosed Between 2006 and 2009: Marginal, Interaction, and Joint Effects

	**PEG study**		**PASIDA study**		**Meta-analysis**	
	Case/control	OR (95% CI)[a]	Case/control	OR (95% CI)[b]	Case/control	OR (95% CI)
Marginal effect						
PRS (per SD)	634/733	1.69 (1.49-1.91)	419/510	1.71 (1.48-2.00	1053/1243	1.70 (1.54-1.87)
TRAP (per IQR)		1.10 (1.04-1.16)		1.11 (0.92-1.35)		1.10 (1.04-1.16)
Multiplicative interaction						
PRS (per SD) x TRAP (per IQR)		1.07 (1.01-1.15)		1.11 (0.85-1.48)		1.07 (1.01-1.14)
Joint effect[c]						
Low PRS x Low TRAP	318/460	1.00	206/311	1.00	524/771	1.00
Low PRS x High TRAP	106/141	1.24 (0.90-1.70)	71/109	1.00 (0.70-1.42)	177/250	1.13 (0.89-1.43)
High PRS x Low TRAP	145/102	2.15 (1.59-2.94)	109/71	2.37 (1.67-3.39)	254/173	2.24 (1.78-2.83)
High PRS x High TRAP	65/30	3.41 (2.13-5.57)	33/19	2.52 (1.38-4.70)	98/49	3.04 (2.08-4.44)
Expected joint effect		2.67 (1.72-4.16)		2.36 (1.43-3.90)		2.53 (1.82-3.53)

Abbreviations: PD, Parkinson disease; PRS, polygenic risk score; TRAP, traffic-related air pollution; SD, standard deviation; IQR, interquartile range; PC, principal component.
[a]Adjusted for age, gender, years of education, study wave, pesticide exposure, smoking status, family history of PD, job history, and 3 PCs of genetic ancestry.
[b]Adjusted for age, gender, education level, smoking status, family history of PD, job history, and 3 PCs of genetic ancestry.
[c]Low (Q1-3) and high (Q4) refer to quartiles of PRS and TRAP exposure.

© 2025 Kwon D et al. *JAMA Network Open*.

eReferences

1. Benson PE. CALINE4 -- A Dispersion Model for Predicting Air Pollutant Concentrations near Roadways, Report No. FHWA/CA/TL-84/15. Office of Transportation Laboratory, California Department of Transportation, Sacramento, CA. http://www.dot.ca.gov/research/researchreports/1981-1988/84-15.pdf
2. Wu J, Laurent O, Li L, Hu J, Kleeman M. Adverse Reproductive Health Outcomes and Exposure to Gaseous and Particulate-Matter Air Pollution in Pregnant Women. *Res Rep Health Eff Inst*. 2016;2016(188):1-58.
3. Wallington TJ, Anderson JE, Dolan RH, Winkler SL. Vehicle Emissions and Urban Air Quality: 60 Years of Progress. *Atmosphere-Basel*. May 2022;13(5)doi:ARTN 65010.3390/atmos13050650
4. Ravi SS, Osipov S, Turner JWG. Impact of Modern Vehicular Technologies and Emission Regulations on Improving Global Air Quality. *Atmosphere-Basel*. Jul 2023;14(7)doi:ARTN 1164 10.3390/atmos14071164
5. Li S, Ritz B, Gong Y, et al. Proximity to residential and workplace pesticides application and the risk of progression of Parkinson's diseases in Central California. *Sci Total Environ*. Mar 15 2023;864:160851. doi:10.1016/j.scitotenv.2022.160851
6. Paul KC, Krolewski RC, Lucumi Moreno E, et al. A pesticide and iPSC dopaminergic neuron screen identifies and classifies Parkinson-relevant pesticides. *Nat Commun*. May 16 2023;14(1):2803. doi:10.1038/s41467-023-38215-z
7. Cockburn M, Mills P, Zhang X, Zadnick J, Goldberg D, Ritz B. Prostate cancer and ambient pesticide exposure in agriculturally intensive areas in California. *Am J Epidemiol*. Jun 1 2011;173(11):1280-8. doi:10.1093/aje/kwr003
8. California Department of Water Resources. Land Use Surveys. (2013)
9. McElroy JA, Remington PL, Trentham-Dietz A, Robert SA, Newcomb PA. Geocoding addresses from a large population-based study: lessons learned. *Epidemiology*. Jul 2003;14(4):399-407. doi:10.1097/01.EDE.0000073160.79633.c1
10. Kusters CDJ, Paul KC, Folle AD, et al. Increased Menopausal Age Reduces the Risk of Parkinson's Disease: A Mendelian Randomization Approach. *Movement Disord*. Oct 2021;36(10):2264-2272. doi:10.1002/mds.28760
11. Ohlei O, Paul K, Nielsen SS, et al. Genome-wide meta-analysis of short-tandem repeats for Parkinson's disease risk using genotype imputation. *Brain Commun*. Jun 11 2024;6(3)doi:ARTN fcae146 10.1093/braincomms/fcae146
12. Nalls MA, Blauwendraat C, Vallerga CL, et al. Identification of novel risk loci, causal insights, and heritable risk for Parkinson's disease: a meta-analysis of genome-wide association studies. *Lancet Neurol*. Dec 2019;18(12):1091-1102. doi:10.1016/S1474-4422(19)30320-5
13. Raaschou-Nielsen O, Taj T, Poulsen AH, et al. Air pollution at the residence of Danish adults, by socio-demographic characteristics, morbidity, and address level characteristics. *Environ Res*. May 15 2022;208:112714. doi:10.1016/j.envres.2022.112714

© 2025 Kwon D et al. *JAMA Network Open*.
