# Supplementary material for: Interaction Between Traffic-Related Air Pollution and Parkinson Disease Polygenic Risk Score
Source: JAMA Netw Open. 2025 Mar 17;8(3):e250854. doi: 10.1001/jamanetworkopen.2025.0854 (PMC11915066; doi:10.1001/jamanetworkopen.2025.0854)
Supplement: Supplement 2. — Data Sharing Statement [file jamanetwopen-e250854-s002.pdf]

Data Sharing Statement

Kwon. Interaction Between Traffic-Related Air Pollution and Parkinson Disease Polygenic Risk Score. *JAMA Netw Open*. Published March 17, 2025.
doi:10.1001/jamanetworkopen.2025.0854

Data
Data available: No

Additional Information
Explanation for why data not available: We are not allowed to share individual level patient data from our research except to collaborators who adhere to our IRB requirements
